# Teachers’ Attitudes Towards STEM Education: Exploring the Role of Their Readiness via a Structural Equation Model

**DOI:** 10.3390/ejihpe14110187

**Published:** 2024-11-04

**Authors:** Theano Papagiannopoulou, Julie Vaiopoulou

**Affiliations:** 1 School of Philosophy and Education, Aristotle University of Thessaloniki, 54124 Thessaloniki, Greece; papagiat@edlit.auth.gr; 2 Department of Education, University of Nicosia, 2417 Nicosia, Cyprus; 3 School of Humanities, Hellenic Open University, 26335 Patras, Greece

**Keywords:** STEM education, teachers’ attitudes, readiness, self-efficacy, commitment, structural equation model

## Abstract

Over the past decade, there has been an intensified emphasis on STEM education to correspond with the goals of twenty-first century education. Educators play a vital role in executing a cohesive approach to interdisciplinary teaching and learning; hence, considerable focus has been directed towards the elements influencing teachers’ attitudes. The study aimed to provide empirical evidence illustrating the significant influence of teachers’ readiness on predicting attitudes. This was achieved by developing a conceptual model that explores the factors affecting individuals’ attitudes towards teaching STEM courses. The emphasis was put on self-efficacy, commitment, cognitive readiness, emotional readiness, and teaching attitudes. A total of 494 Greek primary and secondary education teachers participated electronically in the survey, answering according to the TRi_STEM and TASET scales. The validity of the conceptual model was evaluated using a structural equation model (SEM). The results demonstrated a positive association among all six factors. More notably, general attitudes towards teaching impact positively, either directly or indirectly, the four readiness variables and, finally, teachers’ attitudes towards STEM education. The current study contributes to the existing body of the literature by identifying and analyzing critical attributes that substantially impact teachers’ attitudes towards teaching STEM courses.

## 1. Introduction

To adequately equip kids for the intricate challenges of the twenty-first century, it is imperative to cultivate abilities in science, technology, engineering, and math (STEM) from an early stage, specifically in elementary school [1]. An individual’s inclination towards STEM can be influenced and shaped by early curiosity and relevant experiences. At the same time, prioritizing integrated teaching and learning approaches and teachers’ growth is essential. In order to facilitate the promotion of skills and knowledge about technology, it is necessary to enhance the existing methods, educational goals, and policies of STEM education [2]. There is a growing consensus among members of society regarding the necessity of including STEM knowledge and skill development in the classroom. STEM capabilities refer to the skills that arise when knowledge from separate specialized disciplines and information sources are merged and integrated.

To deal with cultural and work environment shifts using effective and practical technologies, it is urgently necessary to educate children, parents, and instructors on STEM-related challenges [3]. Different STEM practice domains are needed for work, school, and STEM fields [4]; thus, it is crucial to identify the variables influencing teachers’ attitudes towards STEM education as well as the patterns of improvement for its implementation. Therefore, this study aims to detect the readiness factors influencing teachers’ intentions and attitudes regarding the implementation of STEM education. 

Identifying the factors that cause notable teacher attitude variations is advantageous because it allows for developing recommendations to modify these attitudes. This is crucial for successfully executing the required design work, which is vital for effectively implementing the STEM curriculum [5,6]. Several studies have investigated the variables that influence teachers’ attitudes, particularly in order to find the characteristics that have a favorable impact on attitudes [7,8], which are essential for facilitating important learning activities for students’ knowledge. Increased levels of STEM pedagogical topic understanding among educators have been shown to correlate with more positive perceptions of STEM [9]. Furthermore, prior research establishes a correlation between educators’ attitudes towards STEM education and their self-efficacy [9,10]. The emotional health of educators is a crucial factor in cultivating a positive school environment. Educators who reported higher levels of satisfaction in teaching exhibited positive attitudes and allocated additional time for instruction, lesson planning, and evaluating educational objectives [11]. Despite receiving scholarly attention, these determinants have not been collectively evaluated in a singular study. This study seeks to mitigate the existing gap in literature.

### 1.1. Theoretical Considerations About the Variables Included in the Study

#### 1.1.1. Attitudes Towards Teaching

Teachers’ attitudes refer to their emotional and cognitive dispositions that can potentially impact their decisions on instructional practices in the classroom [12]. Individuals’ attitudes towards the teaching profession encompass their perspectives and beliefs about teaching. The effectiveness of educators directly impacts the quality of their students’ learning outcomes [13]. A positive teaching attitude is believed to be associated with professional development, effectiveness, self-confidence, and drive [14,15]. Teachers should exhibit their expertise in the subject matter and unique teaching methodologies, along with their capacity to select suitable instructional materials that align with the class objectives and cater to the needs of their heterogeneous student population.

Moreover, studies indicate a strong association between teachers’ self-efficacy and their inclination to teach [16], and the teachers’ motivation is linked to their willingness to engage in professional development and use learning opportunities. Favorable dispositions towards the teaching profession may enhance motivation throughout professional instruction. These goals can be achieved by promoting a passion for learning, a dedication to achieving excellence, and a consciousness of the profession [15,17].

#### 1.1.2. Cognitive Conditions

Cognitive conditions encompass flexibility, communication, innovative thinking, decision-making, metacognitive methods, pattern identification, problem-solving abilities, resilience, situational awareness, team cohesiveness, and interpersonal skills [18,19]. The cognitive readiness of educators is essential for the implementation of new STEM curricula, given that, if their readiness is inadequate, the execution may falter or be postponed. Instructors must comprehend strategic methodologies and possess the requisite abilities to adapt to educational reforms [20].

Teachers’ pedagogical skills can be evaluated by their ability to skillfully and effectively utilize instructional strategies when implementing STEM activities with students. The teachers’ expertise in their respective subjects forms the basis for the scientific knowledge that students will acquire through STEM activities [21]. Instructors who possess a deep comprehension of STEM pedagogical content exhibit enhanced self-assurance and possess superior proficiency in developing and implementing instructional methodologies [22,23,24]. The lack of clarity in teaching STEM subjects can lead to anxiety when carrying out activities, undermining instructors’ confidence in executing STEM programs and affecting the activities’ quality and effectiveness [22]. The effectiveness of classroom education, the quality of instruction, and the growth of students’ talents are all greatly influenced by a teacher’s cognitive readiness. Teachers who were more knowledgeable and prepared to teach STEM subjects had a positive attitude on the subject [18]. Their self-efficacy is influenced by their insufficient confidence and knowledge necessary to instruct integrated STEM curricula in subject areas that are not directly related to their specialized fields [25,26]. Furthermore, studies have shown that researchers in STEM disciplines demonstrate elevated dedication to their organization, heightened emotional connection, and increased drive to achieve their professional goals compared to non-STEM disciplines [27,28]. Consequently, a number of studies suggest that teachers’ attitudes and intentions regarding STEM education are influenced by their level of readiness [29,30,31].

#### 1.1.3. Self-Efficacy

Personal self-efficacy beliefs impact human behavior the most [32]. The perception of teachers’ self-worth is considered a significant factor that influences their conduct in the classroom, as well as the behavior and learning outcomes of their students. It also influences how others interpret their ideas, behaviors, and emotions in particular situations [33]. Their approach to their work greatly influences teachers’ efficacy and in-class performance. Positive attitudes can foster the creation of a favorable learning environment in the classroom and promote knowledge acquisition [34]. Individuals with high self-efficacy are confident in effectively utilizing instructional tactics in a learning setting, resulting in positive student outcomes [35]. The educators’ assurance and fundamental understanding of incorporating effective STEM teaching often predict the integration of STEM. Moreover, self-efficacy has a direct influence on cognitive capacities. When self-efficacy is stronger, individuals are more likely to set ambitious goals and demonstrate greater commitment to completing specific tasks [36]. Individuals with high self-efficacy consciously opt for student-centered instructional methods, select demanding tasks, demonstrate a strong commitment to achieving their goals, allocate more time and effort to accomplish their objectives [37], and persevere in their endeavors even in the face of failure to meet their personal or organizational goals [38,39]. Individuals with low self-efficacy often stop pursuing opportunities and limit their options due to a lack of confidence in their talents [40]. Consequently, it is presumed that instructors with high levels of self-efficacy will demonstrate increased commitment to their organization and profession. Factors including instructors’ self-efficacy, views regarding academic achievement, emotional pressure, and principal leadership influenced teachers’ motivation and thus the cultivation of a favorable attitude towards STEM instruction [41,42].

#### 1.1.4. Commitment

An educator’s commitment refers to an individual’s emotional attachment or connection with someone or something of high importance or significance [43,44]. Dedication to teaching includes devotion, elevated standards, self-awareness and engagement [45]. Dedicated educators concentrate on their vocation, academic objectives, and ongoing education, hence mitigating burnout and attrition. They affect student performance and development via communication and skill enhancement [44]. Self-efficacy is a critical determinant of dedication, and despite obstacles in executing STEM initiatives, it is essential for sustaining pedagogical methods [46].

Committed educators occasionally have intense emotional attachments to their educational institution, students, or profession. The teaching profession requires remarkable commitment and ongoing innovation to ensure students acquire valuable knowledge. In order for instructors to successfully develop learning activities that incorporate different materials, they must have a deep understanding of the subject matter and possess pedagogical content expertise in one or more STEM domains [47]. Moreover, an array of external and internal factors influences educators’ dedication and contentment, such as personal attributes, administrative direction, the work environment, and societal and economic conditions. Working conditions are linked to emotional exhaustion, work-related anxiety, and fatigue [48].

#### 1.1.5. Affective Conditions

The affective dimension pertains to the influence of emotions on instructors’ capacity to fulfill their duties. The emotions of educators profoundly influence the efficacy of teaching and learning [49]. Emotional exhaustion can result in significant advocates for advancing STEM reform initiatives [50,51]. Emotions are essential for behavioral modification and may serve as a hidden component influencing behavior in a non-linear manner. Consequently, the modification and refinement of professional profiles can facilitate the progression of STEM education [52].

Teaching is a highly emotional undertaking, as educators continually utilize emotions inside and outside the classroom [53,54]. When instructing science and technology courses, students often display adverse emotions such as boredom, trepidation, and uneasiness, which appear to be correlated with their sense of their capacity to achieve. Their self-efficacy beliefs act as a motivating factor for them to participate in tasks where they feel competent and confident. Furthermore, these beliefs significantly influence how much students will dedicate their time and energy to accomplish a task. Similarly, they significantly influence the decisions and behaviors undertaken to comply with these duties [47]. This could result in reduced activities promoting collaborative learning, which may negatively impact students’ general attitudes towards STEM [55]. Additionally, instructors frequently exhibit negative attitudes and emotions towards providing tutoring for STEM courses [56]. This is especially relevant for elementary school educators, who frequently encounter anxiety and feelings of inadequacy due to their limited knowledge of numerous STEM disciplines [57,58,59].

#### 1.1.6. Teachers’ Attitudes Towards STEM Education

The effective implementation of STEM education relies on teachers’ willingness to design instruction outside their specialized areas and their ability to seamlessly integrate relevant engineering and technical concepts into science and math curricula [5,60,61]. Expanding teachers’ training in active teaching methods is crucial for stimulating interest and promoting the growth of scientific professions in their students while also improving their attitudes towards educational practices in STEM domains. Incorporating hands-on activities into expository classes improves the attitudes and emotions of both primary and secondary school students and teachers [56]. Teachers’ attitudes are linked to their teaching practices as they impact their commitment to integrating new concepts into their everyday teaching efforts. The attributes of the teaching environment are crucial as they can either enable or impede the creation of the requisite circumstances for exemplary teaching methodologies [62].

### 1.2. Overview of the Study and the Present Paper

This study aimed to identify the influence of general attitudes towards teaching and teachers’ readiness to implement STEM education on their attitudes towards STEM education. To achieve this goal, 494 Greek teachers participated in the study by completing two self-report questionnaires, i.e., the TRi-STEM scale and the TASET scale. The participants were contacted via email and social media posts.

Six variables were included in the SEM. Attitudes towards teaching acted as the independent variable. The four readiness variables (cognitive conditions, affective conditions, self-efficacy, and commitment) were the mediation factors. Lastly, Attitudes towards STEM education comprised the dependent variable. The research hypotheses were supported by the empirical evidence provided by the analysis.

The present research article comprises the Introduction section, where the included variables are presented in detail, the Materials and Methods section, and the Results section. Lastly, in the Discussion and Conclusions sections the findings are discussed in conjunction with the previous literature, with a special consideration of the limitations and the possible avenues for future research.

## 2. Materials and Methods

### 2.1. The Current Study and the Research Hypothesis

Attitude towards teaching (Att_Teach), cognitive readiness (Cogn), affective readiness (Affe), self-efficacy (SEff), STEM commitment (Comm), and attitude towards STEM education (Att_STEM) are crucial factors that significantly impact the effectiveness and quality of teaching, the successful implementation of the STEM curriculum, and the overall development of educational programs. Although these determinants have received scholarly attention, they have not been analyzed collectively in a single study. According to the literature review conducted before the present study, no existing research collectively examines the relationships among all these variables. Building upon the existing theoretical framework and addressing the existing gap, the present study formulated a the following hypotheses that explore the interactions between the six variables:

**H1.**  *Att_Teach has positive effects on Cogn, Affe, and Att_STEM.*

**H2.**  *SEff has a significant positive relationship with Cogn, Affe, Comm, and Att_STEM.*

**H3.**  *Cogn has a significant positive relationship with SEff, Affe, Comm, and Att_STEM.*

**H4.**  *Comm has positive effects on Affe and Att_STEM.*

**H5.**  *Affe has positive effects on Comm and Att_STEM.*

**H6.**  *Cogn and SEff act as mediators between Att_Teach and Comm.*

**H7.**  *Cogn and SEff act as mediators between Att_Teach and Affe.*

**H8.**  *Affe and Comm act as mediators between Cogn and Att_STEM.*

**H9.**  *Affe and Comm act as mediators between SEff and Att_STEM.*

### 2.2. Participants and Procedures

The study included 494 Greek educators who worked in elementary and secondary schools. The participants’ ages ranged from 43 to 52 (Mean = 44.85, SD = 9.485). The sample consisted of 78.5% female participants. Among the participating teachers, 51.4% held a bachelor’s degree without post-graduate education. Most instructors had between 14 and 26 years of teaching experience (Mean = 17.21, SD = 1.498). In addition, 22.5% of the participants have implemented a relevant program in the educational environment, whereas only 31.2% have taken part in training sessions.

The self-completion questionnaire was submitted electronically through a web form. Following social media and email outreach, the teachers provided their responses anonymously at their own time. All individuals who thoroughly completed the questionnaires were included in the sample of the present study. There were no missing values in the dataset. The enclosed cover letter provided them with information regarding the study’s confidentiality, voluntary participation, and scientific objectives. The employed technique is known as opportunity sampling, and the protocol of has been approved by the Ethics and Deontology Committee of Aristotle University of Thessaloniki, Greece (No. 36728/2023).

### 2.3. Instruments and Measures

The six latent variables under inquiry were represented by two questionnaires (i.e., the TRi-STEM scale and the TASET scale, see below for details) submitted electronically. All the variables were measured on a 9-point Likert scale, where 1 denotes strong disagreement and 9 denotes strong agreement and the participants were asked to rate their level of agreement.

Six variables were included in the SEM. Attitudes towards teaching acted as the independent variable. The four readiness variables (Cognitive conditions, Affective conditions, Self-efficacy, and Commitment) were the mediation factors. Lastly, Attitudes towards STEM education were the dependent variable. The research hypotheses were supported by the empirical evidence provided by the analysis.

#### 2.3.1. Teachers’ Readiness to Implement STEM Education Scale—Tri-STEM Scale

Teachers’ Readiness to Implement STEM Education was measured via the TRi-STEM scale [63], which includes four dimensions, namely cognitive readiness (Cogn), Affective readiness (Aff), STEM commitment (Comm), and Self-efficacy (SEff). The CFA model fit is satisfactory: [*χ*^2^_(249)_ = 981.287, *p* < 0.001, TLI = 0.942, CFI = 0.948, GFI = 0.993, NNFI = 0.942, RMSEA = 0.078 (0.073–0.083), and SRMR = 0.062]. Moreover, the reliability of the measurements using the coefficient Cronbach’s Alpha and McDonald’s Omega for the four factors were calculated as Cogn: *α* = 0.976/*ω* = 0.976, Affe: *α* = 0.972/*ω* = 0.972, Comm: *α* = 0.886/*ω* = 0.885 and Seff: *α* = 0.934/*ω* = 0.935, which suggest a reasonable level of internal consistency for the current measurements using the TRi-STEM scale. Due to the numerical disparity between the genders, measurement invariance has been conducted in four stages (i.e., configural, metric, scalar, strict) in previous research pertaining to the psychometric properties of the TRi-STEM scale, suggesting that the overall model fit is not affected by gender [63]. Consequently, it may be inferred that the outcomes are not significantly influenced by the two genders [64]. Additional information regarding measurement invariance can be located in other sources [65,66,67].

#### 2.3.2. Teachers’ Attitudes Towards STEM Education and Teaching Scale—TASET Scale

Teachers’ attitudes towards STEM education and teaching in general were measured by the TASET scale, a two-dimensional scale that includes Attitudes towards Teaching (Att_Teach) and Attitudes towards STEM (Att_STEM). The scale was adapted based on a work previously published [68] and the items included in the final TASET scale can be found in Table A1 in Appendix A. The CFA model fit is satisfactory: [*χ*^2^_(118)_ = 31.828, *p* < 0.001, TLI = 0.946, CFI = 0.959, GFI = 0.982, NNFI = 0.946, RMSEA = 0.083 (0.074–0.093), and SRMR = 0.054]. Moreover, the reliability of the measurements using the coefficient Cronbach’s alpha and McDonald’s omega for the two factors were calculated as Att_Teach: *α* = 0.901/*ω* = 0.902, and Att_STEM: *α* = 0.952/*ω* = 0.952, respectively, which suggest a reasonable level of internal consistency.

## 3. Results

Table 1 shows the descriptive statistics, means and standard deviations, skewness, and kurtosis of the distributions of each dimension, namely cognitive readiness (Cogn), affective readiness (Affe), STEM commitment (Comm), and self-efficacy (SEff), attitudes towards teaching (Att_Teach) and attitudes towards STEM (Att_STEM). Table 2 presents the correlation matrix of the six dimensions. All correlations were significant at the 0.001 level.

The structural equation modeling (SEM) results are shown in Table A2 (Appendix A) and Table 3, where the factor loadings and the regression coefficients are presented, respectively.

The SEM fit indices were satisfactory, showing a statistically significant model that can describe and explain the associations among the variables predicting teachers’ attitudes towards STEM, Att_STEM [*χ*^2^_(43)_ = 34.767, *p* < 0.001, TLI = 0.940, CFI = 0.946, GFI = 0.931, NNFI = 0.940, RMSEA = 0.059 (0.055–0.062), SRMR = 0.054].

**Figure 1:**
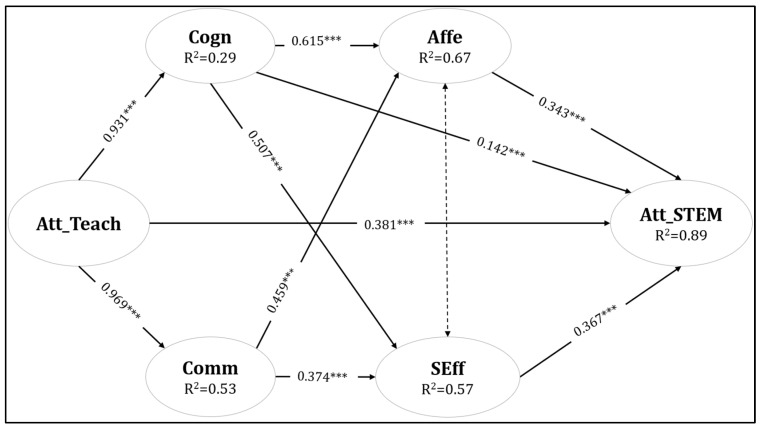
The structural equation model predicting attitudes towards STEM [TLI = 0.940, CFI = 0.946, RMSEA = 0.059, SRMR = 0.054]. Note: Cogn: cognitive readiness, Affe: affective readiness, Comm: STEM commitment, Seff: self-efficacy, Att_Teach: attitudes towards teaching, and Att_STEM: attitudes towards STEM. Note: *** denotes significance at the 0.001 level.

Figure 1 depicts the relationship among the variables used in the SEM.

The model (Figure 1) suggests that there is a direct positive effect from Att_Teach on Att_STEM (*b =* 0.381, *p* < 0.001). An indirect effect via cognitive readiness (Cogn) → affective readiness (Affe) → on Att_STEM, and another indirect effect via fro commitment (Comm) → Self-efficacy (SEF) → on Att_STEM can be observed. An effect from cognitive readiness (Cogn) → on Att_STEM is also present. The mediation analysis showed that all direct and indirect effects under investigation are statistically significant (*p <* 0.001). To this end, the hypothesized SEM proved explanatory to the teachers’ attitudes on STEM education, explaining a large part of the variances [Att_STEM (*R*^2^ = 0.89); SEff (*R*^2^ *=* 0.57); Affe (*R*^2^ = 0.67); Cogn (*R*^2^ = 0.29); Comm (*R*^2^ = 0.53)].

## 4. Discussion

The main aim of this study was to examine the connections between attitudes towards teaching (Att_Teach), cognitive readiness (Cogn) and affective readiness (Affe), Self-efficacy (SEff), STEM commitment (Comm), and attitude towards STEM education (Att_STEM), which are factors that can predict the successful implementation of STEM curriculum. According to the literature, a model was formulated and subsequently subjected to empirical testing. A final model was created using the data of 494 school instructors, which incorporated all indicators and demonstrated satisfactory fit indices.

According to this study, there is a positive correlation between teachers’ attitudes towards their career and their competence, commitment, and attitude towards teaching STEM subjects. Educators who hold constructivist instructional and learning views and have a confident ability to effectively teach integrated STEM courses effectively also demonstrate a positive attitude towards integrated STEM education. Prior studies suggest that instructors who consider a specific teaching and learning strategy as effective are more inclined to demonstrate a favorable attitude towards their teaching practices [69]. Furthermore, the professional and personal development of teachers influences their readiness to modify their classroom practices or behaviors in order to integrate more real-world contexts into their STEM education lessons, demonstrating a greater dedication to providing future opportunities for their students to develop essential STEM skills [70]. Participation in STEM programs significantly improved the personal and professional development of educators by helping them build essential teaching abilities in the area and broaden their understanding of STEM education [5,71,72,73]. Teachers with the necessary knowledge and skills to teach STEM subjects are more inclined to adapt their teaching methods and develop a stronger sense of self-confidence in STEM instruction [74,75]. Novice educators who are unfamiliar with the STEM material often suffer from heightened anxiety and ambiguity around how their expertise and decision-making are perceived by students, families, and colleagues [76]. The current study confirms the impact of cognitive factors on instructors’ self-efficacy.

The study’s results also revealed that the teachers’ level of commitment has a significant and notable impact on their self-efficacy and emotional behavior. The findings are consistent with previous findings [77], which discovered a statistically significant positive relationship between commitment and self-efficacy. Furthermore, research has shown that instructors experience emotional labor due to several personal variables, including personality, teacher self-efficacy, and motivation [78]. This emotional labor also has consequences for the overall well-being of teachers [79,80]. Educators who demonstrate strong enthusiasm and commitment to their work consciously strive to align their emotions and behaviors with their chosen profession. Acknowledging motivation as a pivotal and beneficial element in enhancing professional performance leads to the development of positive and satisfying work-related attitudes [81].

The structural equation modeling (SEM) analysis also revealed a significant correlation between instructors’ perceived self-efficacy and their attitudes towards STEM instruction. Prior research has demonstrated a connection between self-efficacy and instructors’ attitudes, as well as other variables including prior experience and expertise [69,82,83]. Educators that display instructional success typically have a higher level of positivity and openness towards a STEM curriculum. Conversely, teachers who lack self-efficacy show reduced passion for STEM disciplines and may exhibit hesitancy in their teaching approach [84]. This can be explained by the fact that highly effective instructors have a strong belief in their ability to succeed, have deep confidence in their teaching skills, and genuinely enjoy the act of teaching [85]. The viewpoints and importance that educators place on STEM education will impact how they perceive and evaluate newly integrated subjects in the curriculum, as well as their willingness to participate in and apply STEM instruction [86,87]. Furthermore, self-efficacy plays a vital role in forecasting teacher behavior and the effectiveness of educational reform programs [73]. Anxiety emerges when educators feel dissatisfied with their ability to teach effectively, manage their classrooms, or implement educational changes. This leads to a perception of insufficient coping strategies and a lack of confidence in their ability to develop [76].

## 5. Conclusions and Suggestions

This study aimed to investigate the readiness factors associated with teachers’ attitudes towards incorporating Science, Technology, Engineering, and Mathematics (STEM) knowledge and abilities, utilizing a Structural Equation Model (SEM) analysis to examine the connections among these variables. Educators’ attitude towards STEM education was found to be influenced by multiple elements, such as their attitude towards teaching, cognitive conditions, affective conditions, self-efficacy, and STEM dedication. Teachers who deeply understand the basic characteristics, inherent qualities, and teaching methods related to STEM education are more likely to have a favorable attitude towards teaching STEM subjects than teachers who rely mainly on their specialized knowledge in a specific field. The study demonstrates a notable impact of cognitive readiness on STEM attitude, with an indirect impact of cognitive factors on STEM attitude through affective conditions. Confidence and efficacy substantially influence the attitude of instructors in their instructional techniques. The cognitive state of teachers directly impacts on their confidence and belief in their abilities in the classroom, and it also indirectly influences their attitude towards STEM subjects. Our research findings are expected to encourage a more thorough examination of these factors in studies pertaining to teachers’ views. This study has the potential to be a significant reference for future research efforts that aim to investigate the attitudes of STEM teachers.

Policymakers and educators can use this study’s findings to foster STEM education and increase students’ knowledge of the subject and problem-solving skills in an integrated and innovative way [88]. This study helps elementary and secondary schools implement STEM instruction by analyzing teachers’ readiness and attitude. It can help policymakers create meaningful top-level objectives and STEM professional development initiatives for teachers around key impact elements to improve transdisciplinary teaching literacy.

STEM education influences daily tasks and activities; therefore, it is essential for educators to develop STEM projects addressing real-world issues and to recognize the numerous chances for children to investigate, experiment, learn, and propose solutions to everyday problems. Their self-efficacy must be improved because it strongly predicts their attitude. Cognitive and behavioral readiness requires instructors to focus on important requirements, learn from exceptional situations, and analyze them in detail to improve their STEM education and teaching responsibility knowledge. Schools can also host teacher lectures and competitions. To provide teachers with sufficient time to plan for STEM classes, non-teaching responsibilities should be reduced. For more engaging classes, teachers should improve teaching tactics, incorporate STEM subjects into discipline instruction, and provide content for varied student types. Since support, direction, and leadership are essential for instructors to change from traditional teaching techniques, schools should improve STEM education pedagogy and encourage cross-disciplinary collaboration [89]. Finally, facilitating conditions can boost teachers’ teaching intentions and attitude. The state and government can fund or create policies to provide STEM education with instructional venues, resources, technologies, and professional supervision. A STEM network of practice can promote appropriate supporting conditions.

### Limitations

The current study contributes to the existing body of literature by identifying and analyzing critical attributes that substantially impact teachers’ attitudes towards teaching STEM courses. In order to ensure the applicability of these findings to a broader population, it is crucial to replicate them across a broader range of STEM teachers currently employed in different settings, including both elementary and secondary education, and at different points in their career, along with a special consideration to the balance of genders among the participants.

This study identified three limitations. The data collection strategy employed in this study utilized self-report measures, which may have generated a potential bias towards socially desirable responses. As a result, this may have caused a fabricated connection between the factors being assessed. Hence, more inquiries can yield benefits by utilizing various approaches, such as conducting extensive interviews to examine instructors’ perspectives on STEM courses. Moreover, scenario-based evaluations can be utilized alongside teacher-report questionnaires to evaluate educators’ attitudes towards STEM education techniques. To gain deeper insights into the reasons behind educators’ differing levels of self-efficacy and competency in teaching STEM or implementing an integrated STEM curriculum, comprehensive qualitative investigations, including instructor interviews and classroom observations, may be performed.

Another factor that must be considered is the comprehensive evaluation of attitudes towards STEM. Future investigations may employ a more thorough methodology to assess the influence of each of the five measured variables on teachers’ attitudes towards STEM, as well as the interconnectedness among these factors. This research has the potential to provide significant insights into the particular challenges individuals face when trying to implement STEM programs. However, conducting such research necessitates a larger and more comprehensive dataset. Another limitation is the exclusion of environmental variables that may have influenced individuals’ views towards STEM. Subsequent inquiries may integrate environmental factors to improve our understanding of the event. Contextual factors, such as the school environment, might influence individuals’ attitudes towards STEM [90].

## Figures and Tables

**Table 1:** Descriptives, internal consistency indices, skewness, and kurtosis.

		m	SD	α	ω	Skewness	Kurtosis
**Tri-STEM scale**	**Cogn**	5.104	2.271	0.98	0.98	−0.188	−1.073
	**SEff**	4.195	2.025	0.94	0.93	−0.939	0.416
	**Comm**	6.335	1.838	0.89	0.89	−0.151	−1.176
	**Affe**	4.850	2.380	0.97	0.97	0.185	−0.816
**TASET** **scale**	**Att_Teach**	7.097	1.506	0.90	0.90	−1.333	2.140
	**Att_STEM**	5.333	2.244	0.95	0.95	−0.251	−0.992

Note: Cogn: cognitive readiness, Affe: affective readiness, Comm: STEM commitment, Seff: self-efficacy, Att_Teach: attitudes towards teaching, and Att_STEM: attitudes towards STEM.

**Table 2:** Correlation matrix of the six variables included in the model (Pearson’s correlations).

Variable		Cogn	Comm	Affe	SEff	Att_Teach	Att_STEM
**Cogn**	Pearson’s r	—					
	*p*-value	—					
**Comm**	Pearson’s r	0.647	—				
	*p*-value	<0.001	—				
**SEff**	Pearson’s r	0.770	0.642	—			
	*p*-value	<0.001	<0.001	—			
**Att_Teach**	Pearson’s r	0.676	0.533	0.839	—		
	*p*-value	<0.001	<0.001	<0.001	—		
**Att_Teach**	Pearson’s r	0.498	0.668	0.468	0.422	—	
	*p*-value	<0.001	<0.001	<0.001	<0.001	—	
**Att_STEM**	Pearson’s r	0.768	0.657	0.865	0.817	0.604	—
	*p*-value	<0.001	<0.001	<0.001	<0.001	<0.001	—

Note: Cogn: cognitive readiness, Affe: affective readiness, Comm: STEM commitment, Seff: self-efficacy, Att_Teach: attitudes towards teaching, and Att_STEM: attitudes towards STEM.

**Table 3:** SEM regression coefficients.

						95% Confidence Interval		Standardized		
Predictor	Outcome	Estimate	Std. Error	z-Value	*p*	Lower	Upper	All	LV	Endo
Cogn	Affe	0.615	0.052	11.859	<0.001	0.513	0.717	0.556	0.556	0.556
Comm	Affe	0.459	0.070	6.547	<0.001	0.322	0.596	0.320	0.320	0.320
Affe	Att_STEM	0.343	0.068	5.053	<0.001	0.210	0.476	0.366	0.366	0.366
Cogn	Att_STEM	0.142	0.034	4.160	<0.001	0.075	0.209	0.137	0.137	0.137
SEFF	Att_STEM	0.367	0.070	5.214	<0.001	0.229	0.505	0.348	0.348	0.348
Att_Teach	Att_STEM	0.381	0.049	7.761	<0.001	0.285	0.478	0.213	0.213	0.213
	Cogn	0.931	0.088	1.564	<0.001	0.759	1.104	0.540	0.540	0.540
	Comm	0.969	0.084	11.474	<0.001	0.804	1.135	0.728	0.728	0.728
Comm	SEff	0.374	0.072	5.199	<0.001	0.233	0.515	0.293	0.293	0.293
Cogn	SEff	0.507	0.054	9.453	<0.001	0.402	0.612	0.515	0.515	0.515

Note: Cogn: cognitive readiness, Affe: affective readiness, Comm: STEM commitment, Seff: self-efficacy, Att_Teach: attitudes towards teaching, and Att_STEM: attitudes towards STEM.

## Data Availability

The data presented in this study are available upon request from the corresponding author.

## Appendix A

**Table A1:** Teachers’ Attitudes towards STEM Education and Teaching—TASET scale.

PAF with Promax Rotation			Factor Loadings
Attitudes towards STEM (*α* = 0.952, *ω* = 0.952)	SAtt1	I enjoy implementing STEM education.	0.966
	SAtt2	Implementing STEM education is not difficult in my opinion.	0.955
	SAtt3	I have a great time leading the STEM education programs.	0.861
	SAtt4	In STEM education, I set my own educational objectives.	0.832
	SAtt5	I feel ready to implement STEM education.	0.826
	SAtt6	I am constantly looking for the newest information on STEM.	0.812
	SAtt7	I am prepared to take on the obstacles of putting STEM education into practice.	0.832
	SAtt8	I feel confident in my ability to use STEM education.	0.777
	SAtt9	I keep trying to understand how effective STEM education is.	0.739
Attitudes towards Teaching (*α* = 0.901, *ω* = 0.902)	TAtt1	In my daily work as a teacher, I try to identify the underlying causes of challenges.	0.956
	TAtt2	I adapt my teaching strategies to meet the needs of each individual student.	0.938
	TAtt3	I always talk in school about the quality of instruction.	0.719
	TAtt4	I am open to working in conjunction with educators that specialize in different fields than me.	0.710
	TAtt5	I am ready to employ novel teaching methods.	0.642
	TAtt6	In my everyday life, I use the available resources as a teaching tool.	0.612
	TAtt7	I can accept failure during teaching sessions.	0.600
	TAtt8	I am ready to attend seminars about STEM Education.	0.566

CFA model fit: *χ*^2^_(118)_ = 31.828, *p* < 0.001, CFI = 0.959, TLI = 0.946, GFI = 0.982, NNFI = 0.946, RMSEA = 0.083 (0.074–0.093), and SRMR = 0.054.

**Table A2:** SEM factor loadings.

						95% Confidence Interval		Standardized		
Latent	Indicator	Estimate	Std. Error	z-Value	*p*	Lower	Upper	All	LV	Endo
Affective	r.13_Aff_EnjoyImplementingSTEM	1.000	0.000			1.000	1.000	0.907	2.333	0.907
	r.14_Aff_StudentStrengths	0.977	0.026	37.471	<0.001	0.926	1.028	0.901	2.279	0.901
	r.15_Aff_StudentWeaknesses	0.942	0.027	35.389	<0.001	0.890	0.994	0.897	2.198	0.897
	r.16_Aff_TwoWayCommunication	1.007	0.030	33.468	<0.001	0.948	1.066	0.914	2.349	0.914
	r.17_Aff_OrganizedAndSystematic	0.987	0.029	33.908	<0.001	0.930	1.044	0.918	2.302	0.918
	r.18_Aff_GraspOfKnowledge	1.055	0.030	34.669	<0.001	0.995	1.115	0.951	2.461	0.951
Att_STEM	a.36_STEM_ReadyToImplement	1.000	0.000			1.000	1.000	0.823	2.188	0.823
	a.37_STEM_ConfidentToImplement	0.914	0.036	25.243	<0.001	0.843	0.985	0.804	2.001	0.804
	a.42_STEM_GetEfectiveness	0.923	0.045	2.661	<0.001	0.836	1.011	0.824	2.020	0.824
	a.44_STEM_EnjoyLessonsOfTL	1.104	0.042	26.207	<0.001	1.022	1.187	0.912	2.416	0.912
	a.45_STEM_NotDifficultImplement	1.021	0.041	24.748	<0.001	0.940	1.102	0.882	2.234	0.882
	a.47_STEM_LikeImplementation	1.080	0.045	24.064	<0.001	0.992	1.167	0.891	2.362	0.891
	a.48_STEM_SetMyGoals	1.032	0.047	21.980	<0.001	0.940	1.124	0.868	2.257	0.868
	a.50_STEM_MeetChallenges	0.963	0.042	23.099	<0.001	0.881	1.044	0.844	2.106	0.844
	a.55_STEM_LatestSTEMProgram	1.009	0.043	23.713	<0.001	0.926	1.093	0.843	2.208	0.843
Att_Teach	a.34_Teach_TeachersCollaboration	1.000	0.000			1.000	1.000	0.667	1.222	0.667
	a.35_Teach_WorkshopAttendance	1.254	0.074	16.843	<0.001	1.108	1.400	0.681	1.533	0.681
	a.40_Teach_RootCauseInTL	1.135	0.075	15.210	<0.001	0.989	1.281	0.790	1.388	0.790
	a.41_Teach_CustomizeTeaching	1.144	0.074	15.399	<0.001	0.999	1.290	0.800	1.399	0.800
	a.43_Teach_DailyLifeResources	1.196	0.082	14.663	<0.001	1.036	1.356	0.752	1.462	0.752
	a.49_Teach_AcceptFailure	1.083	0.084	12.966	<0.001	0.920	1.247	0.654	1.324	0.654
	a.52_Teach_NewTeachingMethods	1.344	0.087	15.404	<0.001	1.173	1.515	0.798	1.642	0.798
	a.54_Teach_DiscussTeachingQuality	1.119	0.081	13.771	<0.001	0.960	1.279	0.700	1.368	0.700
Cognitive	r.01_Cogn_UnderstandAPSTarget	1.000	0.000			1.000	1.000	0.909	2.108	0.909
	r.02_Cogn_UnderstandTeacherRole	1.021	0.026	39.024	<0.001	0.970	1.072	0.910	2.152	0.910
	r.03_Cogn_UndestandAPS	1.101	0.026	42.278	<0.001	1.050	1.152	0.973	2.321	0.973
	r.04_Cogn_UnderstandAPSDeveloped	1.094	0.026	42.067	<0.001	1.043	1.145	0.972	2.307	0.972
	r.05_Cogn_UnderstandPlannedScope	1.085	0.029	37.423	<0.001	1.028	1.141	0.941	2.286	0.941
Commitment	r.08_Commit_DiscussWithTeachers	1.000	0.000			1.000	1.000	0.703	1.627	0.703
	r.09_Commit_MeaningfulLE	1.261	0.072	17.526	<0.001	1.120	1.402	0.872	2.051	0.872
	r.10_Commit_FunLE	1.203	0.070	17.077	<0.001	1.065	1.341	0.849	1.958	0.849
	r.11_Commit_EffectiveIdeas	0.783	0.057	13.827	<0.001	0.672	0.894	0.594	1.274	0.594
	r.12_Commit_RequirementsMOE	1.024	0.067	15.309	<0.001	0.893	1.155	0.744	1.667	0.744
	r.27_Commit_KnowledgeEnhanceCourses	0.977	0.072	13.520	<0.001	0.836	1.119	0.653	1.590	0.653
Self-Efficacy	r.19_SE_NotFeelBurdened	1.000	0.000			1.000	1.000	0.860	2.076	0.860
	r.20_SE_NotFeelDisappointed	1.019	0.035	28.947	<0.001	0.950	1.088	0.861	2.116	0.861
	r.21_SE_StudentMastery	0.939	0.041	22.752	<0.001	0.858	1.020	0.810	1.950	0.810
	r.22_SE_NotDifficultImplementing	0.950	0.039	24.652	<0.001	0.874	1.025	0.849	1.972	0.849
	r.23_SE_WorkBurden	0.922	0.043	21.390	<0.001	0.837	1.006	0.749	1.914	0.749
	r.30_SE_NotDifficultControlST	0.846	0.041	2.882	<0.001	0.767	0.926	0.769	1.757	0.769
	r.31_SE_EnoughTime	0.771	0.042	18.290	<0.001	0.689	0.854	0.707	1.601	0.707
